# Effect of XlogP and hansen solubility parameters on the prediction of small molecule modified docetaxel, doxorubicin and irinotecan conjugates forming stable nanoparticles

**DOI:** 10.1080/10717544.2021.1958107

**Published:** 2021-07-28

**Authors:** Mei-Qi Xu, Ting Zhong, Xin Yao, Zhuo-Yue Li, Hui Li, Jing-Ru Wang, Zhen-Han Feng, Xuan Zhang

**Affiliations:** a Beijing Key Laboratory of Molecular Pharmaceutics and New Drug Delivery Systems, School of Pharmaceutical Sciences, Peking University, Beijing, China; b Department of Pharmaceutics, School of Pharmaceutical Sciences, Peking University, Beijing, China

**Keywords:** Docetaxel, doxorubicin, irinotecan, hydrophobic parameters (XlogP), solubility parameters, conjugate, nanoparticles, prediction

## Abstract

Small molecule-chemotherapeutic drug conjugate nanoparticles (SMCDC NPs) has a great advantage in improving drug loading. However, the factors which influence these conjugates forming stable nanoparticles (NPs) are currently unclear. In our previous studies, we synthesized a series of fatty acid-paclitaxel conjugates and suggested that the changes in the hydrophobic parameters (XlogP), solubility parameters and crystallinity of these fatty acid-paclitaxel conjugates were the key factors for affecting these small molecule-chemotherapeutic drug conjugates (SMCDCs) forming stable NPs in water. Here, we selected clinically widely used chemotherapeutic drug (docetaxel (DTX), doxorubicin (DOX) and irinotecan (Ir)) as model drug, and chose three straight-chain fatty acids (acetic acid (Ac), hexanoic acid (HA) and stearic acid (SA)) and one branched small molecule (N-(tert-butoxycarbonyl) glycine (B-G)) to synthesize 12 SMCDCs. Our results indicated that our prediction criterions obtained from paclitaxel conjugates were also appropriated for these synthesized SMCDCs. We suggested that the present studies expanded the scope of application of the above-mentioned influencing factors, provided research ideas for the rational design of SMCDC forming NPs and a basis for screening NPs with good anticancer activity.

## Introduction

Poor water solubility is one of the main challenges for clinical applications of chemotherapeutic drugs (Karaosmanoglu et al., 2021). The widely used chemotherapeutic drugs, such as paclitaxel (PTX), doxorubicin (DOX) and irinotecan (Ir), pose a challenge to its clinical application due to their low water solubility. The hydrochloride salt of DOX or Ir can be soluble in water, but excessively high total dose hydrochloride salt solution may cause severe cardiotoxicity and limit its clinical therapeutic efficacy (Mitra et al., 2015).

Nanocarriers have been used in the delivery of chemotherapeutic drugs to reduce their toxic and side effects and enhance their anticancer activity (Piktel et al., 2016; Shi et al., 2017; Pellico et al., 2021). Many nanotechnology-based approaches have been studied for biomedical applications such as liposomes, albumin nanoparticles (NPs) or polymeric micelles. Lipusu® (PTX liposomes), Abraxane^®^, Doxil^®^ (DOX liposomes) and Onivyde^®^ (Ir liposomes) have been approved for clinical treatment. However, the problems such as drug leakage and low drug loading (usually less than 10%) can cause additional systemic toxicity and increase the burden of patient excretion of carrier materials (Shen et al., 2017). Moreover, the existing drug delivery system is too complicated, which is not conducive to clinical transformation.

The emergence of carrier-free NPs that do not rely on carriers has opened up a new path for the construction of stable NPs and the development of anticancer drug platforms, and thus has attracted more attention from researchers (Karaosmanoglu et al., 2021; Sreekanth & Bajaj, 2019; Zheng et al., 2021). The research on macromolecule-drug conjugate NPs started earlier, and some products have successfully entered the clinical trial stage (Guidolin & Zheng, 2019; Peer et al., 2007). Compared with macromolecule-drug conjugate NPs, the methods using small molecule chemotherapeutic drug conjugate nanoparticles (SMCDC NPs) have their own advantages in high drug loading and simple syntheses (Wang et al., 2015). At present, more and more researches are based on SMCDC NPs, such as fatty acid, glycerides, steroids or phospholipid drug conjugates, which can help to improve the pharmacokinetics and biodistribution of drugs, and are used in clinical practice to enhance antitumor efficacy (Sreekanth & Bajaj, 2019; Bhat et al., 2021). The advantages of these SMCDC NPs for cancer chemotherapy monotherapy or combination therapy in combination with photosensitizers, photothermal drugs, immunotherapy drugs or gene drugs have been proven (Huang et al., 2021; Li et al., 2019; Karaosmanoglu et al., 2021). Therefore, research on the rational design, applicability and effectiveness of SMCDC for the formation of NPs is crucial.

In our previous study (Zhong et al., 2018), we designed and synthesized a series of fatty acid-paclitaxel conjugates to study their ability to form NPs, and investigated the influencing factors and *in vitro* anticancer activity. By calculating and analyzing the changes in the hydrophobic parameters (XlogP) and solubility parameters (δd, δp, δh, and δt) of the synthesized paclitaxel (PTX) conjugate compared with PTX, and combining the ability of these synthesized PTX conjugates forming NPs, we proposed the prediction criterions which PTX conjugates can form stable NPs in water: when the XlogP value of PTX conjugates increased by more than 1.0-fold compared to PTX, the conjugate could form stable NPs; otherwise, if its δh and/or δp decreased by more than 10%, the conjugate could also form stable NPs. The prediction criterions were also applicable to judging other PTX conjugates, which has been reported in published literatures, forming stable NPs (Ren et al., 2016). However, the prediction criterions were only obtained from PTX conjugates. We considered that expanding the range of chemotherapeutic drugs was very important for enlarging the application of the prediction criterions.

Here, we selected these three chemotherapeutic drugs (DTX, DOX and Ir) as chemotherapeutic drug models, and chose three straight-chain fatty acids with different chain lengths (acetic acid (Ac), hexanoic acid (HA) and stearic acid (SA)) and one branched small molecule (N-(tert-butoxycarbonyl) glycine (B-G)) to synthesize 12 SMCDCs. In order to verify the feasibility of the prediction criterions obtained from PTX conjugates, in the present research, the XlogP values and solubility parameters of above 12 SMCDCs were calculated, and whether they could form stable NPs was inferred. These SMCDC NPs were prepared by the nano-precipitation method. Through the investigation of particle size, electron microscope and stability, it was verified whether DTX, DOX and Ir conjugates were also suitable for the above inference in order to expand the application scope of the prediction criterions, lifting the limitations of chemotherapeutic drug selection, providing reference and research ideas for the rational design and research of SMCDC NPs in the future. At the same time, studies on the *in vitro* cellular uptake pathways and anticancer efficacy of these conjugate NPs have provided a basis for screening SMCDC NPs with good anticancer activity.

## Materials and methods

### Materials

Docetaxel (DTX), doxorubicin (DOX) and irinotecan (Ir) were obtained from Ouhe Technology Co., Ltd. (Beijing, China). N,N'-dicyclohexylcarbodiimide (DCC), 4-(dimethylamino) pyridine (DMAP), acetic acid (Ac), hexanoic acid (HA), stearic acid (SA) and N-(tert-butoxycarbonyl) glycine (B-G) were purchased from J&K Scientific Ltd. (Beijing, China). Methyl-beta-cyclodextrin (MβCD), hypertonic sucrose and sulforhodamine B (SRB) were obtained from Sigma-Aldrich (St. Louis, MO, USA). All other chemicals were analytical grade or HPLC grade, and used without further purification.

Cell culture medium, RPMI 1640 medium and DMEM medium were obtained from Macgene Biotech Co. Ltd. (Beijing China). Fetal bovine serum (FBS) was supplied from GIBCO (Invitrogen Co. (Carlsbad, USA)).

### Cell lines

Human breast carcinoma MCF-7 cells were obtained from the Cell Resource Center, Peking Union Medial College (Beijing, China). Drug-resistant human breast carcinoma MCF-7/ADR cells were purchased from the Chinese Academy of Sciences Cell Bank (Shanghai, People’s Republic of China). Cells were cultured according to the recommended conditions in ATCC.

### Synthesis of DTX conjugates

The synthesis method of DTX conjugates was the same as the synthesis method of PTX conjugates in previous study (Zhong et al., 2018; Zhong et al., 2016). DTX conjugates were including acetic acid-docetaxel conjugate (Ac-DTX), hexanoic acid-docetaxel conjugate (HA-DTX), stearic acid-docetaxel conjugate (SA-DTX) and N-(tert-Butoxycarbonyl) glycine-docetaxel conjugate (B-G-DTX). In brief, DTX conjugates were synthesized through a direct esterifying reaction on the C2′-hydroxyl of DTX catalyzed by DCC/DMAP. The synthesis route was shown in the figure below. Generally, DTX (80.79 mg, 0.1 mmol) and Ac, HA, SA or B-G (0.1 mmol) were reacted in anhydrous dichloromethane (DCM, 10 ml) containing DCC (41.24 mg, 0.2 mmol) and DMAP (1.22 mg, 0.01 mmol) under nitrogen for 12 h at room temperature. Then, the reaction mixture was filtered to remove N,N'- dicyclohexylurea (DCU) and the filtrate was dried by rotary evaporation. The crude product was purified by silica-gel column chromatography, eluting with methylene chloride/methanol. The resulting eluent was dried by rotary evaporation to obtain a white powder with a yield of 42–55%.


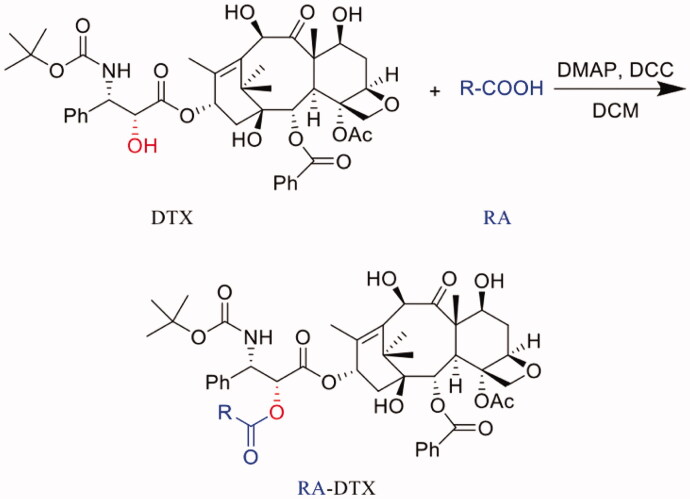


### Synthesis of DOX conjugates

DOX conjugates were synthesized through a condensation acylation reaction catalyzed by HBTU (Wang et al., 2014), including acetic acid-doxorubicin conjugate (Ac-DOX), hexanoic acid-doxorubicin conjugate (HA-DOX), stearic acid-doxorubicin conjugate (SA-DOX) and N-(tert-Butoxycarbonyl) glycine-doxorubicin conjugate (B-G-DOX). The synthesis route was shown in the figure below. Generally, Ac, HA, SA or B-G and HBTU (molar ratio was 1:1.5) were dissolved in the appropriate amount of DMF. After stirring at room temperature for 1 h, doxorubicin hydrochloride (dispersed in DMF in advance) and DIPEA was adding (molar ratio of carboxyl group and amino group was 1: 1). Stirring at room temperature in the dark. After the reaction was completed, the reaction solution was diluted with ethyl acetate, washed with an appropriate amount of dilute hydrochloric acid solution, dilute sodium bicarbonate solution and saturated sodium chloride solution. Then the organic layer was separated to remove water using anhydrous magnesium sulfate. The filtrate was dried under vacuum under reduced pressure to obtain a crude product. The crude product was purified by silica-gel column chromatography, eluting with methylene chloride/methanol. The resulting eluent was dried by rotary evaporation to obtain a dark red powder with a yield of 60–70%.


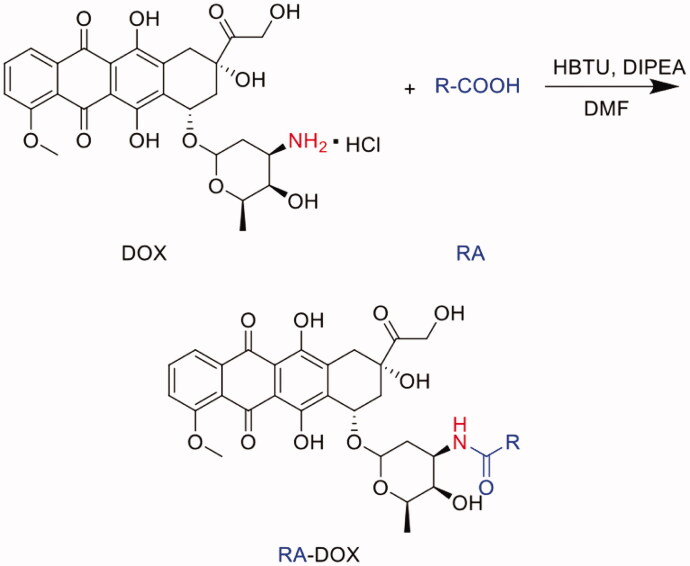


### Synthesis of Ir conjugates

The synthesis method of Ir conjugates was similar to that of PTX conjugates and DTX conjugates, including acetic acid-irinotecan conjugate (Ac-Ir), hexanoic acid-irinotecan conjugate (HA-Ir), stearic acid-irinotecan conjugate (SA-Ir) and N-(tert-Butoxycarbonyl) glycine-irinotecan conjugate (B-G-Ir). The synthesis route was shown in the figure below. Generally, Ir (62.13 mg, 0.1 mmol) and Ac, HA, SA or B-G (0.1 mmol) were reacted in anhydrous dichloromethane (DCM, 10 ml) containing DCC (41.24 mg, 0.2 mmol) and DMAP (1.22 mg, 0.01 mmol) under nitrogen for 12 h at room temperature. The obtained residue was reconstituted with an appropriate amount of ethyl acetate, washed with dilute sodium bicarbonate solution and saturated sodium chloride solution. Then the organic layer was separated and anhydrous magnesium sulfate was added to remove water. The filtrate was dried under vacuum under reduced pressure to obtain a crude product. The crude product was purified by silica-gel column chromatography, eluting with methylene chloride/methanol. The resulting eluent was dried by rotary evaporation to obtain a pale yellow powder with a yield of 40–50%.


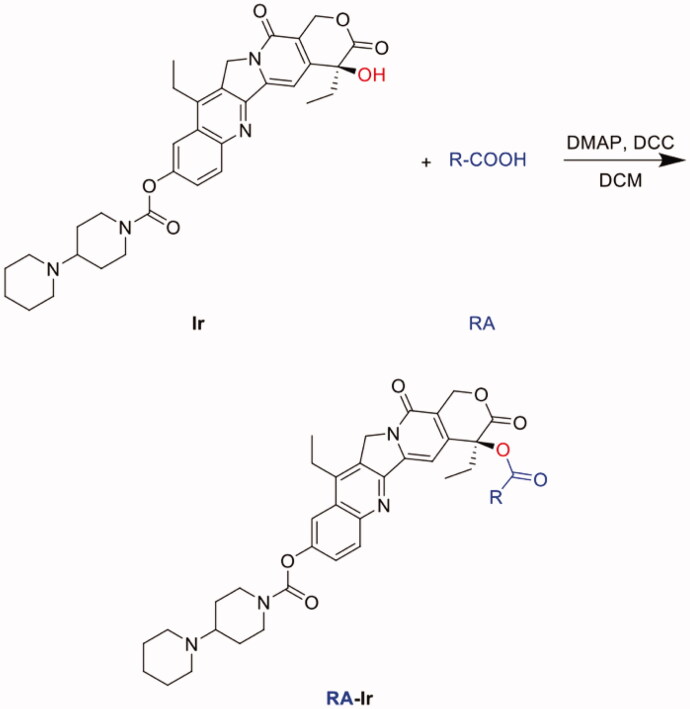


### Characterization of conjugates

**NMR spectra**. The conjugates were dissolved in deuterated chloroform and determined by nuclear magnetic resonance spectroscopy (400 MHz ^1^H NMR and ^13 ^C NMR, Bruker AVANCEZ 400).

**Crystallinity**. The crystallinity was examined by powder X-ray diffraction (XRD) using a D/MAX 2000 rotating anode X-ray diffractometer (Rigaku Co., Japan) equipped with a Cu-Kα X-ray source (λ = 1.541 nm, 40 kV/100 mA). All the XRD data were collected over a 2θ range from 3° to 40° at a step size of 0.02° at increments for quantitative analysis. Profile fits of the data were performed using Origin 8.5.1.

**Polarity**. The polarity of all samples was evaluated by high performance liquid chromatography (HPLC), using an LC-20AT liquid chromatograph (SHIMADZU, Japan) and SPD-M20A diode array detector (SHIMADZU, Japan). The determination of these conjugates was carried out on an ODS analytical column (Luna^®^ 5 μm C18 (2), 250 × 4.60 mm, 5 μm; Phenomenex, Torrance, CA, USA) with a column temperature of 40 °C.

### Theoretical partition coefficient: XlogP value

The calculation method of theoretical partition coefficient was shown in our previous report (Zhong et al., 2018). Briefly, XLOGP3 developed by Wang group (Cheng et al., 2007) was used to calculate the theoretical partition coefficient of each compound. ChemBio3D Ultra 2010 software was used to construct a three-dimensional structure model of each compound. All final models were saved as calculated input data in Tripos Mol2 format.

### Hansen solubility parameters

According to the GCM proposed by Beerbower (Hansen, 2007), the polar solubility parameter (δp), and hydrogen bonding solubility parameter (δh) were calculated respectively as shown in our previous study (Zhong et al., 2018). The calculation details were shown in Table S1-3.

### Preparation and characterization of NPs

All the conjugate NPs were prepared by nanoprecipitation, as shown in our previous report (Zhong et al., 2016). The conjugate and DSPE-PEG (conjugate: DSPE-PEG = 1:0.1, w/w) was dissolved in DMSO to obtain a concentration of the conjugate of 30 mg/ml. 1 ml of this solution was added drop-wise to 3 ml of distilled water with continuous and gentle stirring (300–500 rpm) at room temperature. Under these conditions, the conjugate NPs occurred spontaneously. Then, the obtained NP dispersion was dialyzed against distilled water for 48 h (MWCO = 3500 Da) , and subjected to ultra-filtration at 4000 rpm for 5 min. The concentration of conjugate in the obtained conjugate NPs was 6 mg/ml (adjusted with distilled water).

The particle size, particle size distribution, and zeta potential were measured by dynamic light scattering (DLS). A transmission electron microscope (TEM) (JEM-1400Plus, JEOL Ltd. Tokyo, Japan) was used to examine the morphology of the NPs.

### *In vitro* cytotoxicity

The cytotoxicity of conjugate NPs was evaluated against MCF-7 or MCF-7/ADR cells using a sulfonyl rhodamine B (SRB) colorimetric assay. Generally, MCF-7 or MCF-7/ADR cells were seeded in 96-well plates at a density of 6 × 10^3^ cells/well, and adhered at 37 °C for 24 h. Then, cells were exposed to increasing concentrations of NPs. After 48 h incubation, the culture medium was discarded, and the post-treatment method was the same as that in the previous study (Zhang et al., 2019).

### The endocytosis pathway of NPs

Hypertonic sucrose (inhibitor of clathrin-dependent endocytosis) and MβCD (inhibitor of caveolin-dependent endocytosis) were selected to study the internalization mechanisms of the conjugate NPs. MCF-7/ADR cells were plated in 12-well tissue culture plates at 2 × 10^5^ cells per well for 24 h proliferation. Then, the cell culture medium was replaced with serum-free medium containing 0.4 M hypertonic sucrose or 10 mM MβCD, incubated for 30 minutes. Then, the NPs at the final concentration of 10 μM were exposed to the cells and incubated for an additional 4 h at 37 °C. After incubation, cells were washed three times with cold PBS (pH 7.4), and lysed with 0.1% SDS (0.1 ml). A volume of 5 μl of the lysate were taken for the determination of the protein concentration using a Pierce^TM^ BCA Protein Assay Kit (Thermo Scientific, Waltham, USA). Then, the lysate was extracted with 0.3 ml acetonitrile, and centrifuged at 10000 rpm for 10 min. A volume of 20 μl of the resulting supernatant was used for measuring the content of drug through HPLC analysis. Three wells were measured for each sample. The cellular uptake was calculated using the following formula:

$$\text{Cellular uptake }=\frac{\text{The concentration of drug in the cells of each well}}{\text{The concentration of total protein in the cells of each well}}$$

### Statistical analysis

All experimental data are expressed as the mean ± SD. The significance among groups was determined using one-way analysis of variance (ANOVA) with Bonferroni *post hoc* correction for comparisons between individual groups. Statistical significance was shown at *p* < .05.

## Results and discussion

### Effect of XlogP and hansen solubility parameters on the formation of DTX conjugate NPs

In our previous study (Zhong et al., 2018), we proposed the prediction criterions which PTX conjugates could form to stable NPs in water: when the XlogP value of PTX conjugates increased more than 1.0-fold compared to PTX, the conjugate could form stable NPs; otherwise, if its δ_h_ and/or δ_p_ decreased more than 10%, the conjugate could also form stable NPs. DTX has a similar structure to PTX and two-fold greater antimitotic efficacy than PTX. It can be used to treat lung, breast and ovarian cancer (Choi & Park, 2017). In order to verify whether the prediction criterions established in the previous study was suitable for predicting the ability of DTX conjugates to form NPs, we designed four DTX conjugates, including Ac-DTX, HA-DTX, SA-DTX and B-G-DTX.

**Figure 1.**
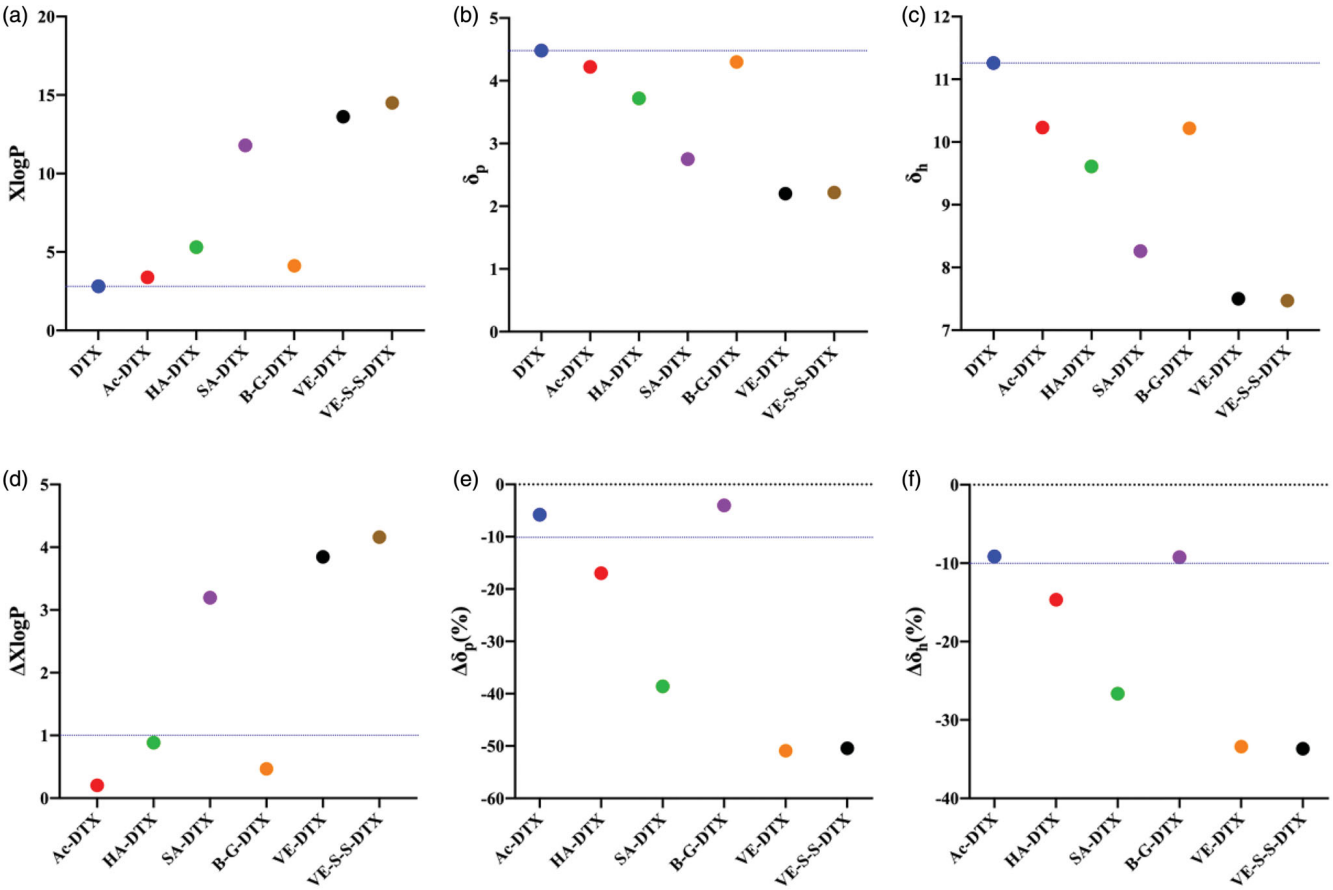
Effect of XlogP and Hansen solubility parameters on DTX conjugate NPs. (a) XlogP values of DTX and DTX conjugates calculated using XLOGP3; (b) Polar solubility parameter (δ_p_) values of DTX and DTX conjugates according to Beerbower; (c) Hydrogen bonding solubility parameter (δ_h_) values of DTX and DTX conjugates according to Beerbower; (d) ΔXlogP values of DTX conjugates, ΔXlogP = (XlogP value of DTX conjugates − XlogP value of DTX)/XlogP value of DTX. (e) Δδ_p_ values of DTX conjugates, Δδ_p_ values = (δ_p_ value of DTX − δ_p_ value of DTX conjugates) × 100%/δ_p_ value of DTX); (f) Δδ_h_ values of DTX conjugates, Δδ_h_ values = (δ_h_ value of DTX − δ_h_ value of DTX conjugates) × 100%/δ_h_ value of DTX).

XLOGP3 was used to calculate the theoretical hydrophobic parameter (XlogP) values of DTX and its conjugates respectively. The results showed that the XlogP values of all designed DTX conjugates were higher than that of DTX (Figure 1(a)). Among them, the XlogP value of the straight-chain fatty acid-DTX conjugate significantly increased with the length of the modified carbon chain, that was, DTX (2.81) <Ac-DTX (3.38) <HA-DTX (5.30) <SA-DTX (11.79), and the XlogP value of B-G-DTX (4.12) was between Ac-DTX and HA-DTX. In addition, we also calculated the XlogP values of the reported DTX conjugates which could form stable NPs, including vitamin E-disulfide bond-DTX conjugate (VE-SS-DTX) and vitamin E-DTX conjugate (VE-DTX) (Ren et al., 2016). The results showed that the XlogP values of these two reported DTX conjugates were 14.50 and 13.62, which were significantly higher than DTX, and also higher than those of our designed four DTX conjugates (Figure 1(a)).

Then, the group contribution method (GCM) was used to calculate the Hansen solubility parameters (δ) of DTX and its conjugates, including polar solubility parameters (δp) and hydrogen bond solubility parameters (δh). The results showed that the δp and δh values of four designed DTX conjugates were significantly lower than those of DTX and decreased with the length of fatty acid carbon chain (Figure 1(b,c)). In addition, the δp and δh values of the reported DTX conjugates VE-SS-DTX and VE-DTX (Ren et al., 2016) were significantly lower than DTX, and also lower than those of our designed four DTX conjugates.

From the above results, the ΔXlogP value and ΔHansen solubility parameters of our designed DTX conjugates and reported DTX conjugates were calculated (Figure 1(d–f)). The ΔXlogP value of SA-DTX increased more than 1.0-fold compared with DTX, which met our prediction criterions for forming stable NPs. So we speculated that SA-DTX could form stable NPs in water. The XlogP values of other DTX conjugates such as Ac-DTX, HA-DTX and BG-DTX did not increase more than 1.0-fold compared with DTX, so the percentage of δp and δh reduction (Δδp (%) and Δδh (%)) need to be further considered. The Δδp (%) and Δδh (%) of HA-DTX exceeded 10%, while the Δδp (%) and Δδh (%) of Ac-DTX and B-G-DTX was less than 10%. Therefore, we speculated that HA-DTX could also form stable NPs, while Ac-DTX and B-G-DTX could not form stable NPs in water (could not form NPs or form NPs with poor stability). In addition, the ΔXlogP values of the reported DTX conjugates VE-S-S-DTX and VE-DTX (Ren et al., 2016) increased more than 1.0-fold compared with DTX, which also met the criterions proposed in the previous study.

**Figure 2.**
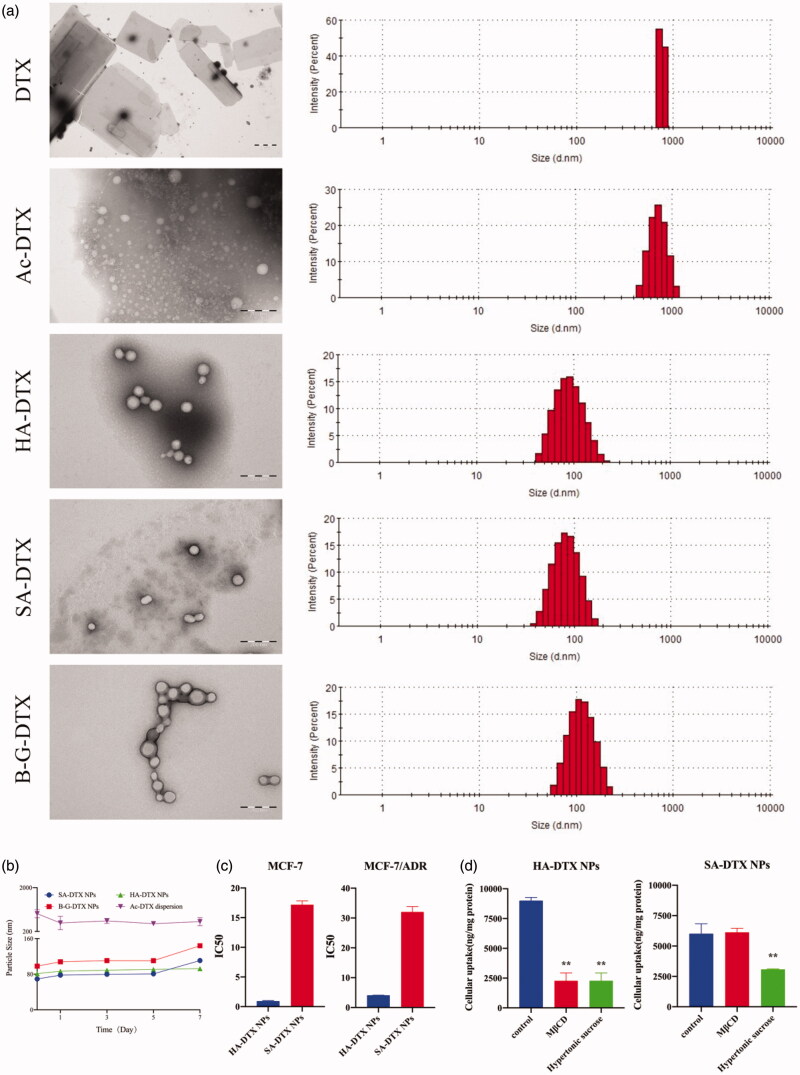
The particle size, transmission electron microscopy (TEM), *in vitro* anticancer activity and endocytosis pathways of DTX conjugate NPs. (a) TEM images and particle size of DTX conjugate NPs; (b) Changes in particle size of DTX conjugate NPs during a week; (c) IC_50_ of HA-DTX NPs and SA-DTX NPs in MCF-7 and MCF-7/ADR cell lines; (d) The endocytosis pathways of HA-DTX NPs and SA-DTX NPs on MCF-7/ADR cell line.

In order to verify above speculation, we synthesized these four designed DTX conjugates, and their characterization were shown in Figure S1, S4 and S7. Then, these DTX conjugate NPs were prepared by the nano-precipitation method. The results of particle size and transmission electron microscopy (TEM) experiments confirmed that the HA-DTX and SA-DTX could form uniform monodisperse spherical NPs (Figure 2(a)), which were consistent with the above speculation. DTX formed lamella-like crystals, and Ac-DTX formed random aggregates. The B-G-DTX could also form NPs, however, a small amount of precipitate was deposited after 48 h placement (Figure 2(b)). Therefore, the ability of DTX conjugates to form NPs predicted by ΔXlogP and Δδh and Δδp was confirmed by our designed DTX conjugates, which HA-DTX and SA-DTX could form stable NPs, while Ac-DTX and B-G-DTX could not.

### *In vitro* anticancer activity and endocytosis pathways of DTX conjugate NPs

Because HA-DTX and SA-DTX could form stable NPs, the *in vitro* anticancer activity of HA-DTX NPs and SA-DTX NPs was investigated in MCF-7 and MCF-7/ADR cell lines respectively. As shown in Figure 2(c), in both MCF-7 and MCF-7/ADR cell lines, the IC_50_ value of HA-DTX NPs was lower than that of SA-DTX NPs. We suggested that this was because the greater the XlogP value of DTX conjugate, the better the stability of NPs and the more difficult to dissolve. Therefore, after NPs were absorbed into the tumor cells, they still existed in NPs form without dissolving and releasing the conjugate molecules, resulting in a decrease in anticancer activity. On this basis, we believed that the balance of XlogP value for designed conjugates should be considerable, otherwise, the antitumor activity of the conjugate NPs could be decreased. When designing a hydrophobic DTX conjugate NP, the length of the modified chain should be shortened as much as possible (XlogP was reduced) on the basis of the formation of NPs, which was more conducive to its anticancer activity.

Endocytosis inhibitors were used to investigate the endocytosis pathways of DTX conjugate NPs in MCF-7/ADR cells, as shown in Figure 2(d). The results showed that MβCD significantly inhibited the cellular uptake of HA-DTX NPs (*p < 0.01*), and had no significant effect on the cellular uptake of SA-DTX NPs (*p > 0.05*). Hypertonic sucrose significantly inhibited the cellular uptake of SA-DTX NPs (*p < 0.01*), and had no significant effect on the cellular uptake of HA-DTX NPs (*p > 0.05*). Therefore, SA-PTX NPs were taken up into cells through clathrin-dependent endocytosis. HA-DTX NPs could be taken up into cells through caveolin-dependent endocytosis. Different DTX conjugate NPs had different cellular uptake pathways, which might lead to different anticancer activity.

### Effect of XlogP and hansen solubility parameters on the formation of DOX conjugate NPs

**Figure 3.**
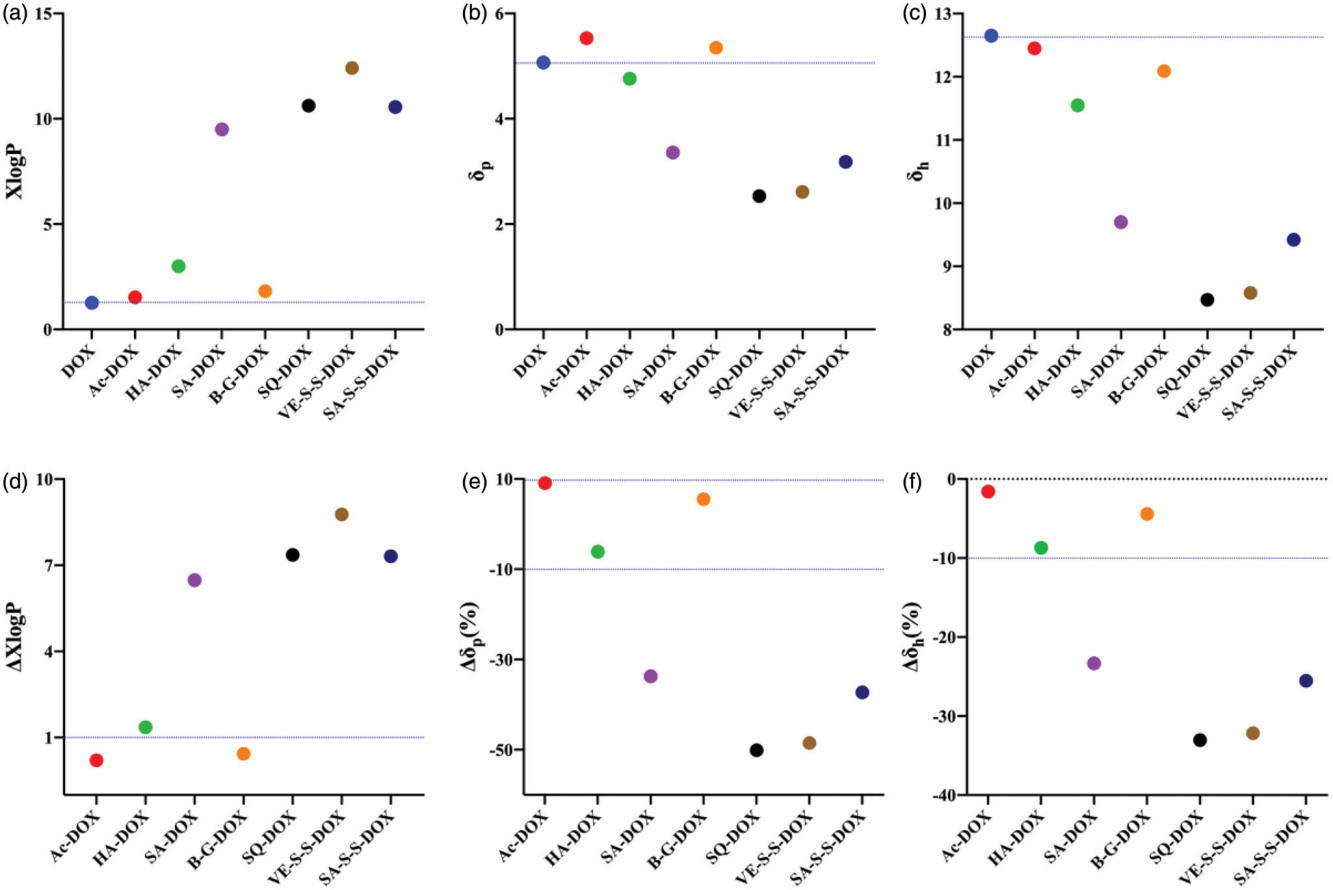
Effect of XlogP and Hansen solubility parameters on DOX conjugate NPs. (a) XlogP values of DOX and DOX conjugates calculated using XLOGP3; (b) Polar solubility parameter (δ_p_) values of DOX and DOX conjugates according to Beerbower; (c) Hydrogen bonding solubility parameter (δ_h_) values of DOX and DOX conjugates according to Beerbower; (d) ΔXlogP values of DOX conjugates, ΔXlogP = (XlogP value of DOX conjugates − XlogP value of DOX)/XlogP value of DOX. (e) Δδ_p_ values of DOX conjugates, Δδ_p_ values = (δ_p_ value of DOX − δ_p_ value of DOX conjugates) × 100%/δ_p_ value of DOX); (f) Δδ_h_ values of DOX conjugates, Δδ_h_ values = (δ_h_ value of DOX − δ_h_ value of DOX conjugates) × 100%/δ_h_ value of DOX).

DOX can inhibit the synthesis of nucleic acids by intercalating between the double-stranded bases of DNA to inhibit the synthesis of nucleic acids. It is a widely used anticancer chemotherapeutic drug in clinical practice (Mitra et al., 2015; Pattni et al., 2015). We also tried to use DOX to construct NPs, and further investigated whether the prediction criterions established in the previous study was suitable for predicting the ability of DOX conjugates to form NPs. Similarly, we designed Ac-DOX, HA-DOX, SA-DOX and B-G-DOX. The XlogP value and Hansen solubility parameters (δ) of DOX and its conjugates were calculated separately. As shown in Figure 3(a), the XlogP value of DOX conjugates increased significantly with the length of the fatty acid carbon chain. The δp and δh values of four designed DOX conjugates were significantly lower than those of DOX and decreased with the length of fatty acid carbon chain (Figure 3(b,c)). By calculating ΔXlogP, we found the XlogP value of SA-DOX and HA-DOX increased more than 1.0-fold compared with DOX (Figure 3(d)), so it was speculated that both SA-DOX and HA-DOX could form stable NPs. However, the ΔXlogP values of Ac-DOX and B-G-DOX were not more than 1, we further considered the Δδp and Δδh of Ac-DOX and B-G-DOX. As shown in Figure 3(e), the Δδp (%) and Δδh (%) of Ac-DOX and B-G-DOX were still less than 10%. Therefore, we speculated that Ac-DOX and B-G-DOX could not form stable NPs in water (could not form NPs or form NPs with poor stability). At the same time, we calculated the ΔXlogP value of reported DOX conjugates, such as SQ-DOX (Maksimenko et al., 2014), VE-SS-DOX (Wang et al., 2014) and SA-SS-DOX (Wang et al., 2014), which could form stable NPs. Our results indicated that XlogP values of above reported DOX conjugates were more than 1.0-fold compared with DOX (Figure 3(d)), showing that our proposed criterions are also suitable for predicting above DOX conjugates forming stable NPs.

**Figure 4.**
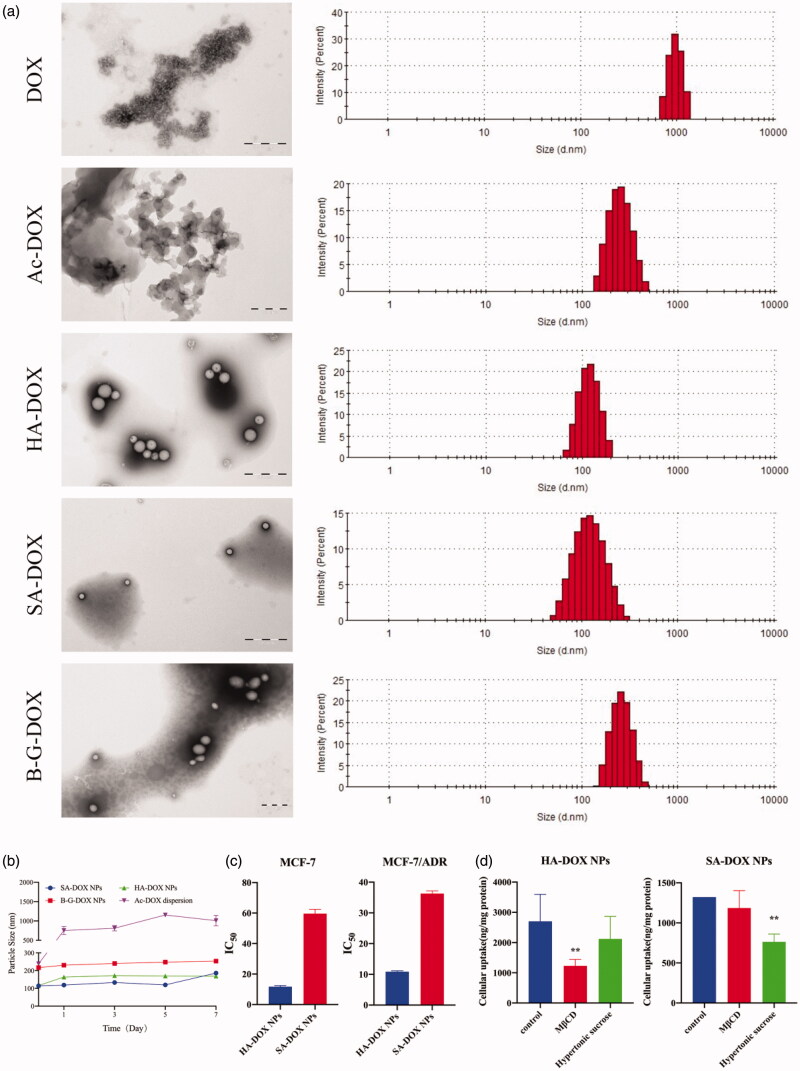
The particle size, transmission electron microscopy (TEM), *in vitro* anticancer activity and endocytosis pathways of DOX conjugate NPs. (a) TEM images and particle size of DOX conjugate NPs; (b) Changes in particle size of DOX conjugate NPs during a week; (c) IC_50_ of HA-DOX NPs and SA-DOX NPs in MCF-7 and MCF-7/ADR cell lines; (d) The endocytosis pathways of HA-DOX NPs and SA-DOX NPs on MCF-7/ADR cell line.

Then, we synthesized these four designed DOX conjugates, and their characterizations were shown in Figures S2, S5 and S8. Next, the nano-precipitation method was used to verify the ability of DOX conjugates to form NPs. As shown in the Figure 4(a,b), the results were consistent with the previous criterions, which HA-DOX and SA-DOX could form stable NPs, while Ac-DOX and B-G-DOX could not. Therefore, the prediction criterions applicable to predicting PTX and DTX conjugates forming stable NPs was also applied to predict DOX conjugates forming stable NPs.

### *In vitro* anticancer activity and endocytosis pathways of DOX conjugate NPs

MCF-7 and MCF-7/ADR cell lines were also selected to study the anticancer activity of HA-DOX NPs and SA-DOX NPs *in vitro*. As shown in Figure 4(c), in both MCF-7 and MCF-7/ADR cell lines, the IC_50_ value of HA-DOX NPs was lower than that of SA-DOX NPs. This was because the greater the XlogP value of DOX conjugate, the better the stability of NPs and the more difficult to dissolve. Therefore, when designing a hydrophobic DOX conjugate NP, the length of the modified chain also should be shortened as much as possible (XlogP was reduced) on the basis of the formation of NPs, which was more conducive to its anticancer activity.

The endocytosis pathways of HA-DOX NPs and SA-DOX NPs in MCF-7/ADR cells were shown in Figure 4(d). The results showed that SA-DOX NPs were taken up into cells through clathrin-dependent endocytosis. HA-DOX NPs could be taken up into cells through caveolin-dependent endocytosis.

### Effect of XlogP and hansen solubility parameters on the formation of Ir conjugate NPs

**Figure 5.**
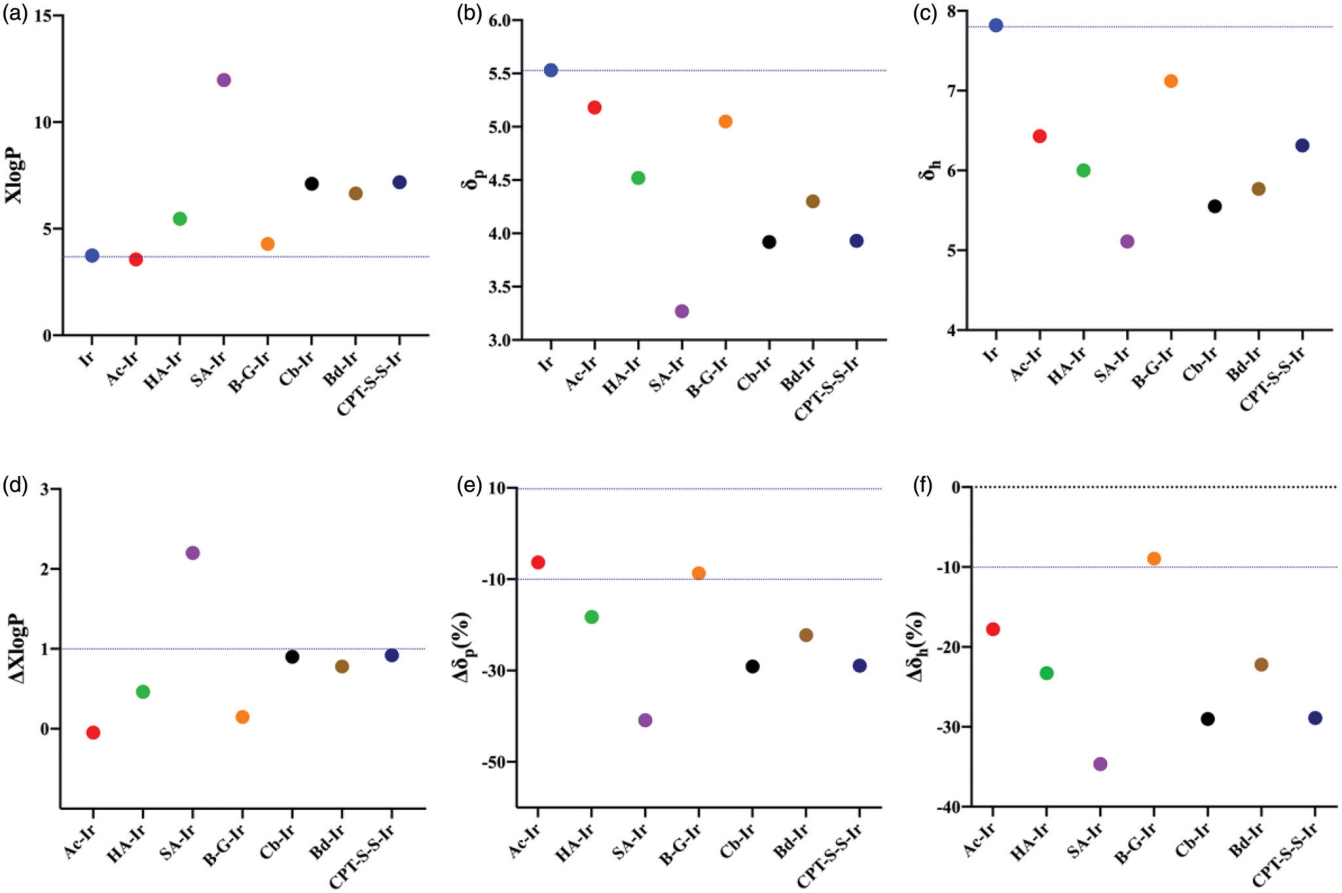
Effect of XlogP and Hansen solubility parameters on Ir conjugate NPs. (a) XlogP values of Ir and Ir conjugates calculated using XLOGP3; (b) Polar solubility parameter (δ_p_) values of Ir and Ir conjugates according to Beerbower; (c) Hydrogen bonding solubility parameter (δ_h_) values of Ir and Ir conjugates according to Beerbower; (d) ΔXlogP values of Ir conjugates, ΔXlogP = (XlogP value of Ir conjugates − XlogP value of Ir)/XlogP value of Ir. (e) Δδ_p_ values of Ir conjugates, Δδ_p_ values = (δ_p_ value of Ir − δ_p_ value of Ir conjugates) × 100%/δ_p_ value of Ir); (f) Δδ_h_ values of Ir conjugates, Δδ_h_ values = (δ_h_ value of Ir − δ_h_ value of Ir conjugates) × 100%/δ_h_ value of Ir).

Irinotecan (Ir) is a semi-synthetic derivative of camptothecin, and itself and its active metabolite SN-38 can be combined with topoisomerase I-DNA complex, thereby preventing the reconnection of broken single strands, causing DNA double strand breaks. It has a certain anticancer activity in most tumors. Currently, it is mainly used in the treatment of digestive system-related tumors such as gastric cancer, colon cancer and rectal cancer (Fujita et al., 2015). Then, we selected Ir as a model drug to design Ac-Ir, HA-Ir, SA-Ir and B-G-Ir, and further investigated whether the prediction criterions we established was suitable for predicting the ability of Ir conjugates to form NPs. The XlogP, δ, ΔXlogP and Δδ values of four our designed Ir conjugates and three reported Ir conjugates which could form stable NPs (chlorambucil-irinotecan conjugate (Cb-Ir) (Huang et al., 2014), bendamustine-irinotecan conjugate (Bd-Ir) (Huang et al., 2015) and camptothecin-disulfide bond-irinotecan conjugate (CPT-SS-Ir) (He et al., 2016)) were calculated separately (Figure 5). By calculating ΔXlogP, we found the ΔXlogP of SA-Ir was more than 1, so it was speculated that SA-Ir could form stable NPs (Figure 5(d)). The ΔXlogP of Ac-Ir, HA-Ir and B-G-Ir were not more than 1, so we further considered the Δδp (%) and Δδh (%) of them. As shown in Figure 5(e), the Δδp (%) and Δδh (%) of HA-Ir were more than 10%, and the Δδp (%) and Δδh (%) of Ac-Ir and B-G-Ir were less than 10%. Therefore, we speculated that SA-Ir and HA-Ir could form stable NPs, Ac-Ir and B-G-Ir could not form stable NPs in water (could not form NPs or form NPs with poor stability). In addition, although the ΔXlogP of these three reported Ir conjugates were less than 1, their Δδp (%) and Δδh (%) were both greater than 10%, which indicated that the criterions we proposed were also suitable for predicting the formation of reported Ir conjugate NPs.

**Figure 6.**
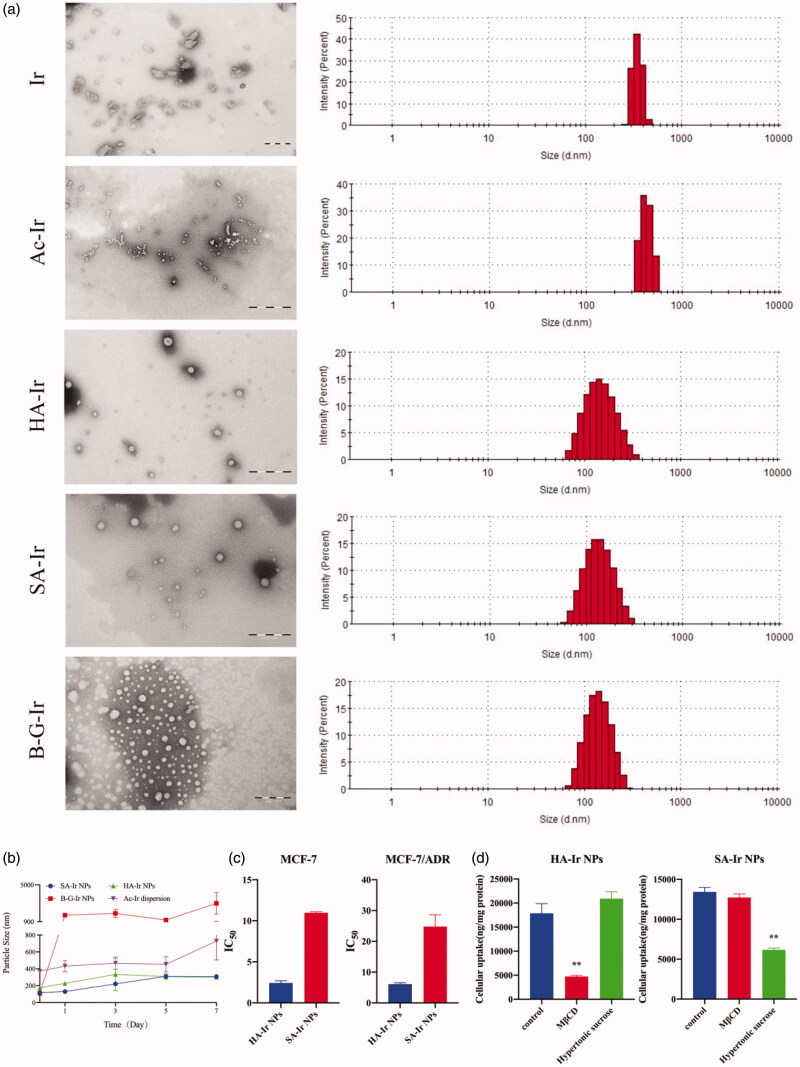
The particle size, transmission electron microscopy (TEM), *in vitro* anticancer activity and endocytosis pathways of Ir conjugate NPs. (a) TEM images and particle size of Ir conjugate NPs; (b) Changes in particle size of Ir conjugate NPs during a week; (c) IC_50_ of HA-Ir NPs and SA-Ir NPs in MCF-7 and MCF-7/ADR cell lines; (d) The endocytosis pathways of HA-Ir NPs and SA-Ir NPs on MCF-7/ADR cell line.

Then, we synthesized these four designed Ir conjugates (Ac-Ir, HA-Ir, SA-Ir and B-G-Ir), and their characterization were shown in Figure S3, S6 and S9. The nano-precipitation method was used to verify the ability of Ir conjugates to form NPs. As shown in the Figure 6(a,b), the results were consistent with the previous speculation, which HA-Ir and SA-Ir could form stable NPs, while Ac-Ir and B-G-Ir could not. Although B-G-Ir could also form NPs, in the stability experiment, there was a small amount of precipitation of the B-G-Ir dispersion within 24 h (Figure 6(b)). Therefore, the prediction criterions applicable to predicting PTX, DTX and DOX conjugates forming stable NPs was also applied to predict Ir conjugates forming stable NPs.

### *In vitro* anticancer activity and endocytosis pathways of Ir conjugate NPs

*In vitro* anticancer activity of HA-Ir NPs and SA-Ir NPs were studied in MCF-7 and MCF-7/ADR cell lines (Figure 6(c)). The results showed that the IC_50_ value of HA-Ir NPs was lower than that of SA-Ir NPs, which was consistent with DTX conjugates and DOX conjugates. The high XlogP value made conjugates NPs more stable and difficult to dissolve and release, resulting in reduced anticancer activity.

The endocytosis pathway of HA-Ir NPs and SA-Ir NPs in MCF-7/ADR cells was shown in Figure 6(d). The results showed that SA-Ir NPs were taken up into cells through clathrin-dependent endocytosis. HA-Ir NPs could be taken up into cells through caveolin-dependent endocytosis. Combining the results of the endocytosis pathways of DTX, DOX and Ir conjugate NPs, we found that HA-modified conjugate NPs were all absorbed through clathrin-mediated endocytosis, while SA-modified conjugate NPs were all absorbed through caveolin-dependent endocytosis. Therefore, we suggested that the uptake of conjugate NPs was closely related to the small-molecule fatty acids that modify chemotherapeutic drugs. When designing conjugate NPs, we could consider changing the uptake pathway of NPs by changing the modified small molecules so that NPs could reach different organelles to exert their anticancer activity.

In recent years, the arsenal of nanomedicine platforms are expanding rapidly. Liposomal cytarabine–daunorubicin (Vyxeos; also known as CPX-351) was approved for marketing by the FDA (Alfayez et al., 2020). Liposomal cisplatin (Lipoplatin) (Stathopoulos et al., 2010), liposomal paclitaxel (EndoTAG-1) and polymeric micelle paclitaxel (NK105) (Fujiwara et al., 2019) have entered the phase III clinical stage. Polymeric NPs of DTX (BIND-014) (Autio et al., 2018) and polymeric NPs of camptothecin (CRLX‐101) (Clark et al., 2016) have entered the phase II clinical stage. Liposomes, along with polymeric micelle and NPs, still account for a large part of clinical stage nanotherapeutics. Although it has been widely shown that encapsulating drugs in liposomes or micelles can improve pharmacokinetics (PK) and biodistribution, when directly compared with traditional chemotherapeutic drugs, there are no commercially available therapeutic agents that have an overall survival advantage (Alfayez et al., 2020). The loading efficiency of chemotherapeutic drugs is still very low, and their long-term toxicity is unclear. The development of carrier-free NPs is a promising solution. These NPs are made of pure drug molecules and do not involve any organic or inorganic carriers. The carrier-free NPs have the higher delivery efficiency and the better safety. Therefore, the research to explore the formation law of carrier-free NPs has high research value. There are few studies on how to rationally design SMCDC NPs. Our research expanded the application scope of the prediction criterions of previously proposed PTX conjugates, provided reference and research ideas for the rational design and construction of SMCDC NPs.

In the present study, we constructed SMCDC NPs which directly bind through ester bonds. Some studies reported prodrugs which connected by flexible linkers (such as disulfide bonds) could form stable NPs. We chose some reported conjugates which directly bind through ester bonds (OA-PTX (Zhong et al., 2018), VE-DTX (Ren et al., 2016) and SA-DOX) or conjugates which connected by flexible linkers disulfide bonds (OA-S-S-PTX (Luo et al., 2016), VE-S-S-DTX (Ren et al., 2016) and SA-S-S-DOX (Wang et al., 2014)) and calculated their XlogP values and ΔXlogP values. As shown in Table S4, the results showed that the ΔXlogP values of these conjugates which connected by flexible linker disulfide bonds were ​​greater than that of directly bind through ester bonds, indicating that disulfide bond increased the XlogP value of the conjugate. The increased XlogP made the formation of NPs easier, so the existence of disulfide bonds could contribute to forming stable NPs. In addition, all the ΔXlogP values, both of obtained from directly bind through ester bond conjugates and from disulfide bond conjugates, met our prediction criterions.

Our previous results indicated that the *in vitro* antitumor activity of SMMDC NPs was reduced along with the increased XlogP values (Zhong et al., 2018). A suitable XlogP value for designing SMMDCs is important for forming NPs and for possessing antitumor activity. However, for disulfide bond conjugates, their XlogP values were relative high. These disulfide bonds could respond to the tumor microenvironment to release the parent drug, the XlogP value of which was relatively lower than that of SMMDC. Therefore, when designing a SMCDC NP, it is possible to shorten the length of the modified chain (XlogP was reduced) on the basis of the formation of stable NPs, or to introduce sensitive bonds in the conjugate to enhance its antitumor efficacy.

## Conclusion

For the prediction criterions of the previously proposed PTX conjugates could form stable NPs in water, here, we used DTX, DOX and Ir as model drugs, and chose three straight-chain fatty acids with different chain lengths and one branched small molecule to synthesize 12 SMCDCs. By comparing the predicted ability to form NPs with the actual ability to form NPs, an attempt was made to expand the scope of the prediction criterions of PTX conjugates. We reported that whether it was a DTX conjugate, DOX conjugate or Ir conjugate, they all met the prediction criterions proposed before, namely: the XlogP value of the conjugates should increase more than 1.0-fold compared with that of the parent drug; otherwise, their Hansen solubility of the polar solubility parameter (δp) and hydrogen bond solubility (δh) should decrease more than 10% compared with the parent drug. In addition, on the basis of the formation of stable NPs, the length of the modified chain was shortened as much as possible, which was more conducive to its anticancer activity.

In summary, this study expanded the scope of the prediction criterions whether SMCDC can form stable NPs by using hydrophobic parameters (XlogP) and solubility parameters, so as to rationally design and construct SMCDC NPs provide references and research ideas. At the same time, it also provided a basis for screening SMCDC with good anticancer activity.

## Supplementary Material

Supplemental Material
