# Supplementary material for: Effect of XlogP and hansen solubility parameters on the prediction of small molecule modified docetaxel, doxorubicin and irinotecan conjugates forming stable nanoparticles
Source: Drug Deliv. 2021 Jul 28;28(1):1603–15. doi: 10.1080/10717544.2021.1958107 (PMC8330778; doi:10.1080/10717544.2021.1958107)
Supplement: Supplemental Material [file IDRD_A_1958107_SM5138.docx]

**Effect of XlogP and Hansen Solubility Parameters on the Prediction of Small Molecule Modified Docetaxel, Doxorubicin and Irinotecan Conjugates forming stable nanoparticles**

Mei-Qi Xu^a,b^ **^$^**, Ting Zhong^a,b^ **^$^**, Xing Yao^a,b^, Zhuo-Yue Li^a,b^, Hui Li^a^, Jing-Ru Wang^a,b^, Zhen-Han Feng^a,b^, Xuan Zhang ^a,b^ *

a Beijing Key Laboratory of Molecular Pharmaceutics and New Drug Delivery Systems, School of Pharmaceutical Sciences, Peking University, Beijing 100191, China

b Department of Pharmaceutics, School of Pharmaceutical Sciences, Peking University, Beijing 100191, China

^$^These authors contributed equally to this work

* Corresponding author: [xuanzhang@bjmu.edu.cn](mailto:xuanzhang@bjmu.edu.cn)

* Corresponding author:

Dr. Xuan Zhang

Professor

[xuanzhang@bjmu.edu.cn](mailto:xuanzhang@bjmu.edu.cn)

Department of Pharmaceutics,

School of Pharmaceutical Sciences,

Peking University,

Xueyuan Road 38,

Beijing 100191, China

Tel: +86-10-82805765

Fax: +86-10-82805765

**Supporting Information**

**Calculation of solubility parameter and molar volume**

**Table S1.** Calculation of solubility parameter and molar volume for DTX.

| Docetaxel（DTX） |  | | | | | |
| --- | --- | --- | --- | --- | --- | --- |
| Structure groups | number of groups | F_d_  (J^1/2^ cm^3/2^ mol^-1^) | F_p_  (J^1/2^ cm^3/2^ mol^-1^) | F^2^_p_  (J cm^3^ mol^-2^) | E_h_  (J mol^-1^) | V  (cm^3^ mol^-1^) |
| -CH_3_ | 8 | 419 | 0 | 0 | 0 | 31.70 |
| -CH_2_- | 3 | 270 | 0 | 0 | 0 | 16.60 |
| >CH- | 8 | 80 | 0 | 0 | 0 | -1.00 |
| >C< | 5 | -70 | 0 | 0 | 0 | -19.20 |
| =C< | 2 | 45 | 70 | 4900 | 143 | -5.70 |
| 5 or 6 member ring | 2 | 190 | 0 | 0 | 0 | 13.50 |
| -CONH- | 1 | 516 | 1270 | 1612900 | 5985 | 16.80 |
| -OH | 4 | 211 | 499 | 249001 | 9773 | 10.47 |
| >C=O | 1 | 291 | 769 | 591361 | 978 | 10.00 |
| -COO-ester | 3 | 667 | 511 | 261121 | 2557 | 8.20 |
| -O- | 2 | 235 | 409 | 167281 | 2352 | 3.60 |
| Phenyl- | 2 | 1499 | 121 | 14641 | 205 | 75.40 |
| Σ |  | 12042 |  | 4357272 | 59126 | 466.28 |
| δ_p_ | 4.48 J^1/2^ cm^-3/2^ | | | | | |
| δ_h_ | 11.26 J^1/2^ cm^-3/2^ | | | | | |

**Table S2.** Calculation of solubility parameter and molar volume for DOX.

| doxorubicin  (DOX) |  | | | | | | | | |
| --- | --- | --- | --- | --- | --- | --- | --- | --- | --- |
| Structural group | Number  of groups | F_d_  (J^1/2^ cm^3/2^ mol^-1^) | | F_p_  (J^1/2^ cm^3/2^ mol^-1^) | F^2^_p_  (J cm^3^ mol^-2^) | | E_h_  (J mol^-1^) | | V  (cm^3^ mol^-1^) |
| -CH_3_ | 2 | 419 | | 0 | 0 | | 0 | | 31.70 |
| -CH_2_- | 4 | 270 | | 0 | 0 | | 0 | | 16.60 |
| >CH- | 5 | 80 | | 0 | 0 | | 0 | | -1.00 |
| >C< | 1 | -70 | | 0 | 0 | | 0 | | -19.20 |
| 5 or 6 member ring | 3 | 190 | | 0 | 0 | | 0 | | 13.50 |
| Conjugation ring | 3 | 43 | | 0 | 0 | | -227 | | 0.80 |
| -NH_2_ | 1 | 370 | | 419 | 175561 | | 3220 | | 17.93 |
| -OH | 5 | 211 | | 499 | 249001 | | 9773 | | 10.47 |
| >C=O | 3 | 291 | | 769 | 591361 | | 978 | | 10.00 |
| -O- | 2 | 235 | | 409 | 167281 | | 2352 | | 3.60 |
| -O- aromatic | 1 | 100 | | 401 | 160801 | | 1467 | | 3.80 |
| Phenyl< | 2 | 1319 | | 133 | 17689 | | 205 | | 60.40 |
| Σ |  | 8453 |  | | | 3725390 | | 60919 | 380.58 |
| δ_p_ | 5.07 J^1/2^ cm^-3/2^ | | | | | | | | |
| δ_h_ | 12.65 J^1/2^ cm^-3/2^ | | | | | | | | |

**Table S3.** Calculation of solubility parameter and molar volume for Ir.

| Irinotecan (Ir) |  | | | | | |
| --- | --- | --- | --- | --- | --- | --- |
| Structural group | Number  of groups | F_d_  (J^1/2^ cm^3/2^ mol^-1^) | F_p_  (J^1/2^ cm^3/2^ mol^-1^) | F^2^_p_  (J cm^3^ mol^-2^) | E_h_  (J mol^-1^) | V  (cm^3^ mol^-1^) |
| -CH_3_ | 2 | 419 | 0 | 0 | 0 | 31.70 |
| -CH_2_- | 13 | 270 | 0 | 0 | 0 | 16.60 |
| >CH- | 1 | 80 | 0 | 0 | 0 | -1.00 |
| >C< | 1 | -70 | 0 | 0 | 0 | -19.20 |
| =CH- | 1 | 223 | 70 | 4900 | 143 | 12.40 |
| =C< | 6 | 45 | 70 | 4900 | 143 | -5.70 |
| 5 or 6 member ring | 6 | 190 | 0 | 0 | 0 | 13.50 |
| Conjugation ring | 3 | 43 | 0 | 0 | -227 | 0.80 |
| >N- | 1 | 31 | 149 | 22201 | 366 | -9.00 |
| -N= | 1 | 164 | 1323 | 1750329 | 1759 | 4.00 |
| -CON< | 2 | 301 | 1229 | 1510441 | 4772 | 13.20 |
| -OH | 1 | 211 | 499 | 249001 | 9773 | 10.47 |
| -COO-ester | 1 | 667 | 511 | 261121 | 2557 | 8.20 |
| -O- aromatic | 1 | 100 | 401 | 160801 | 1467 | 3.80 |
| Phenyl< | 1 | 1319 | 133 | 17689 | 205 | 60.40 |
| Σ |  | 9214 |  | 5516324 | 25991 | 424.87 |
| δ_p_ | 5.53 J^1/2^ cm^-3/2^ | | | | | |
| δ_h_ | 7.82 J^1/2^ cm^-3/2^ | | | | | |

**Table S4** Effect of S-S on XlogP value and ΔXlogP of conjugates

| Compound | XlogP | ΔXlogP |
| --- | --- | --- |
| OA-PTX | 11.71 | 2.20 |
| OA-S-S-PTX | 12.23 | 2.34 |
| VE-DTX | 13.62 | 3.85 |
| VE-S-S-DTX | 14.50 | 4.16 |
| SA-DOX | 9.5 | 6.48 |
| SA-S-S-DOX | 10.56 | 7.31 |

**NMR spectra of conjugates**


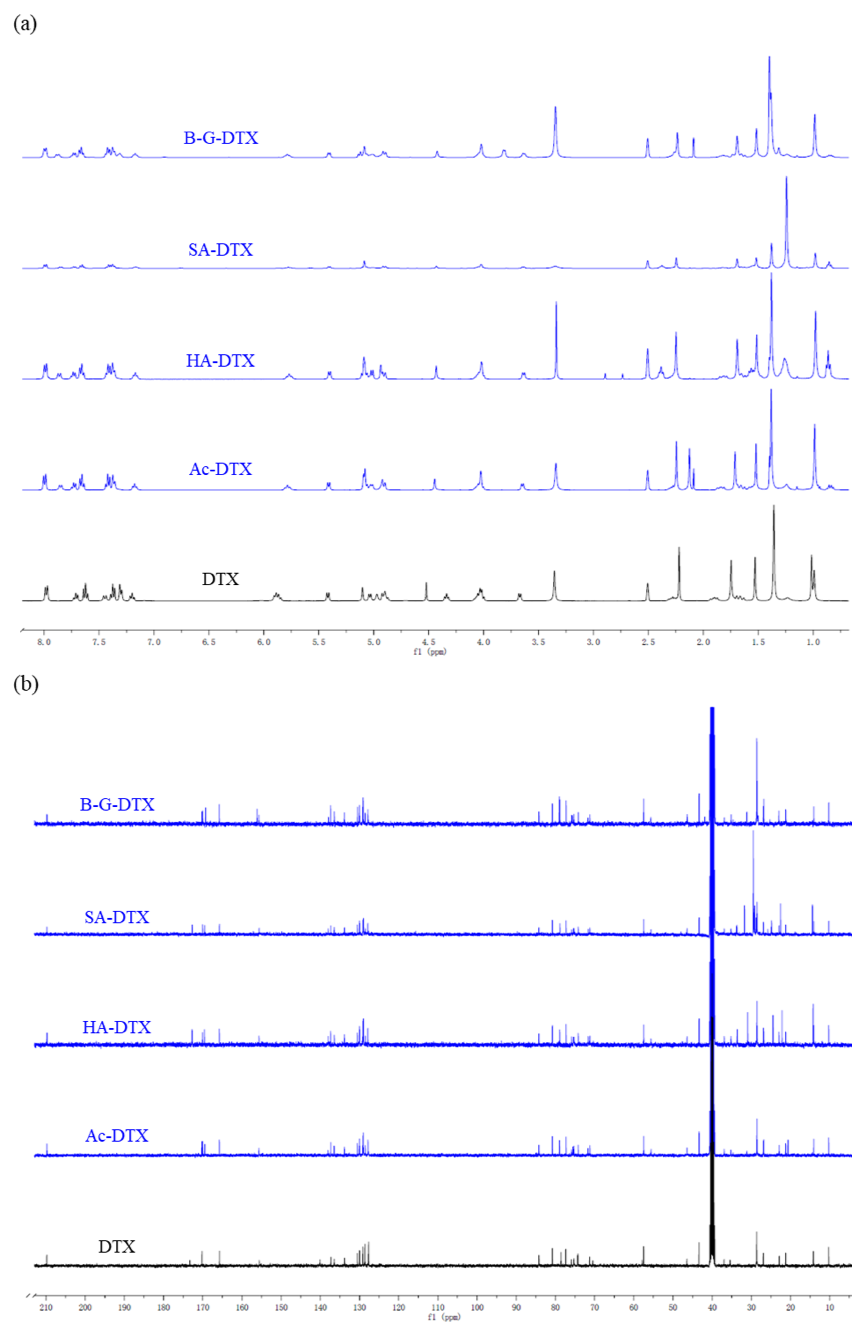


#### **Figure S1**. NMR spectra of DTX conjugates. (a) ^1^H NMR spectra; (b) ^13^C NMR spectra.


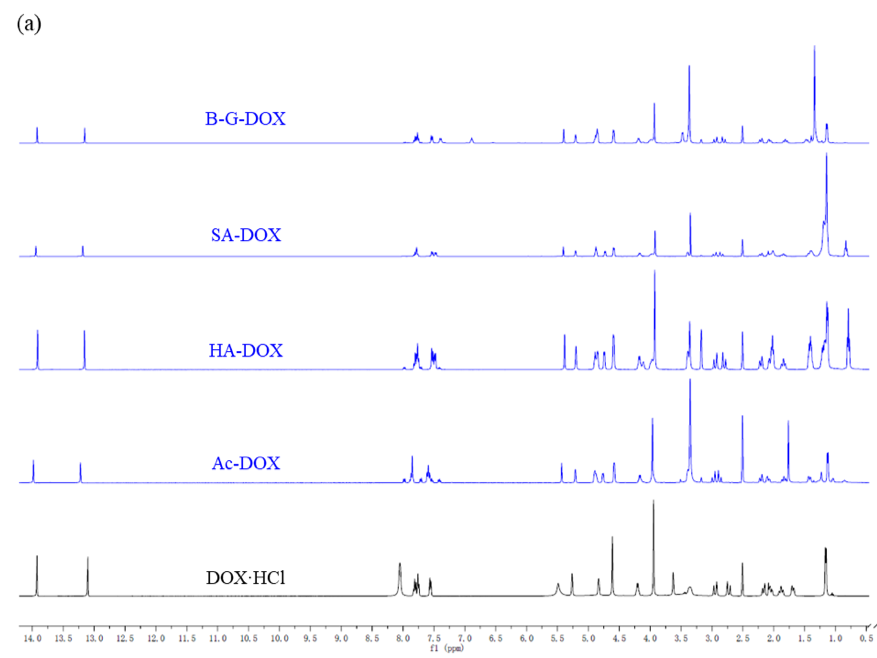

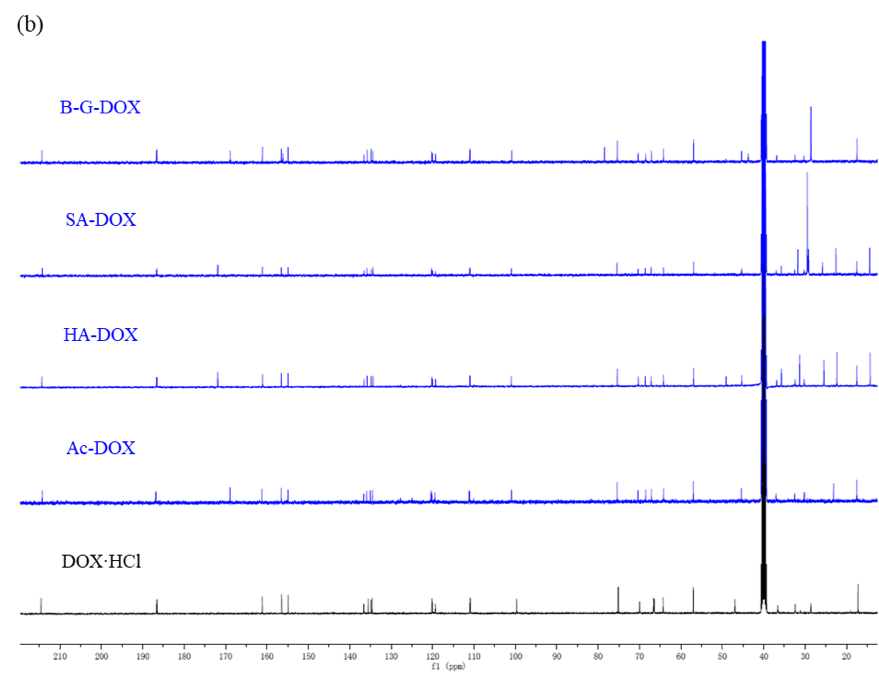


#### **Figure S2.** NMR spectra of DOX conjugates. (a) ^1^H NMR spectra; (b) ^13^C NMR spectra.


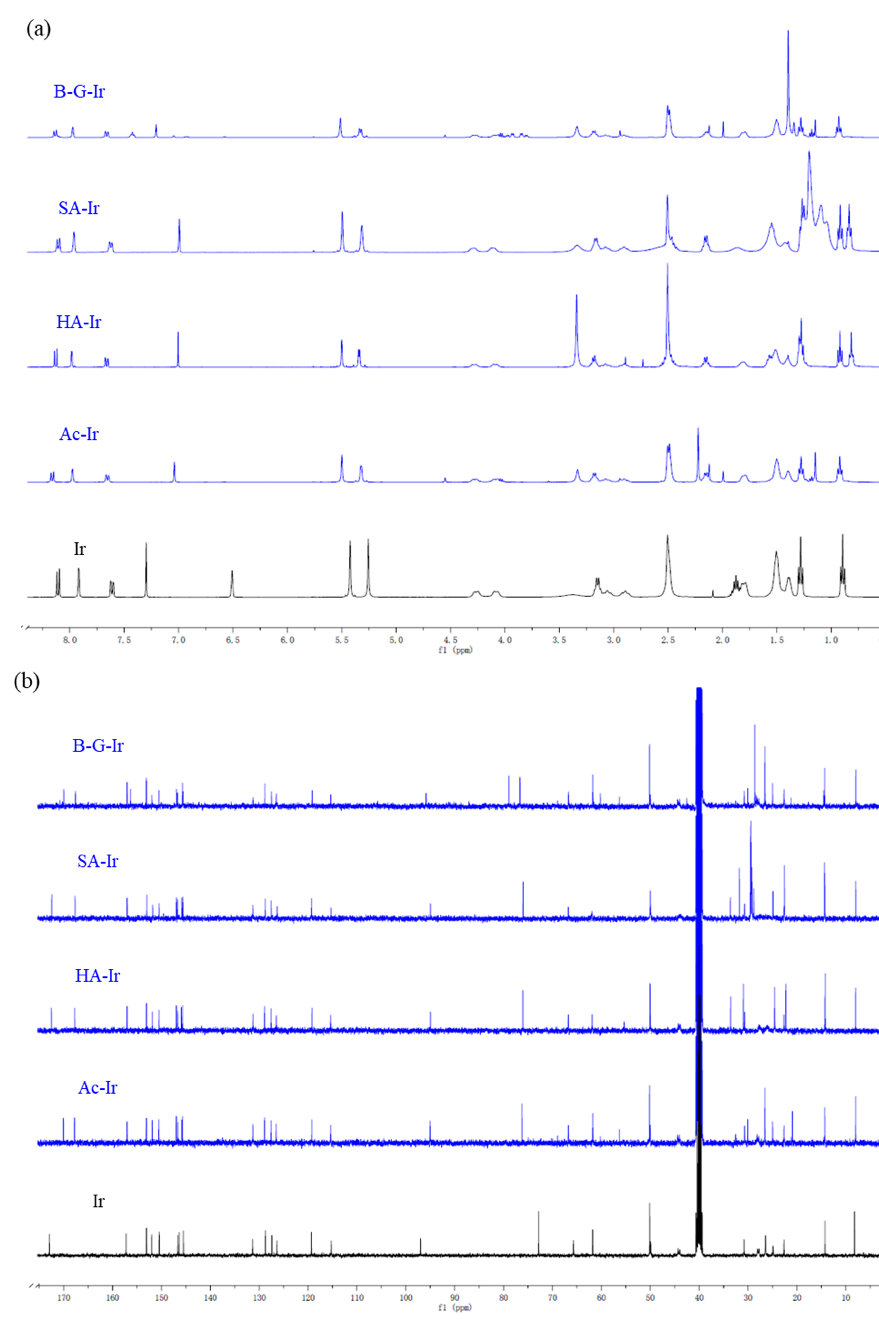


#### **Figure S3.** NMR spectra of Ir conjugates. (a) ^1^H NMR spectra; (b) ^13^C NMR spectra.

**HPLC chromatogram of conjugates**


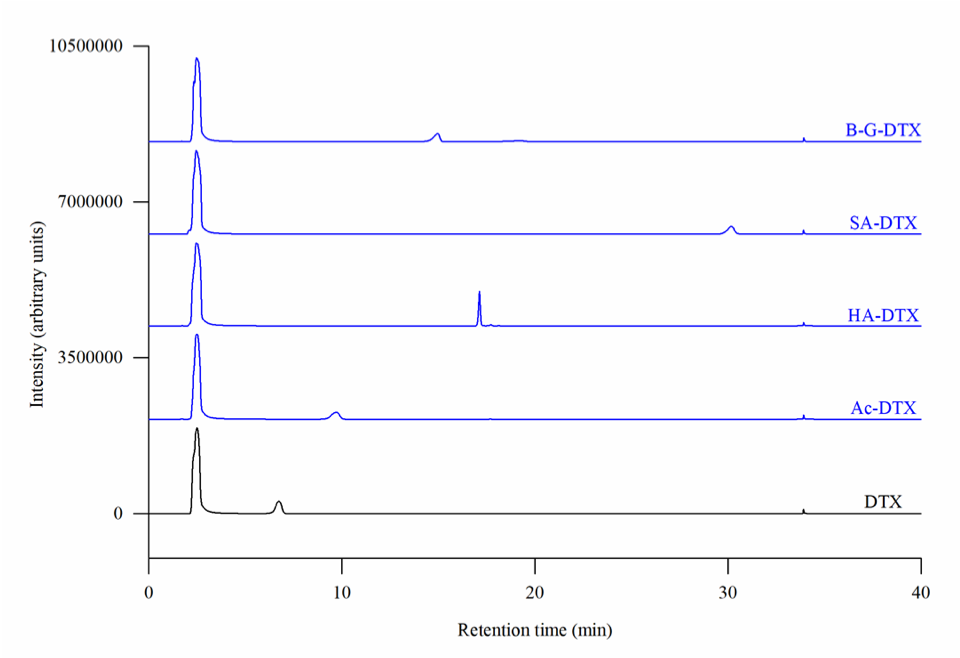


**Figure S4. HPLC** **chromatogram of DTX conjugates.** The DTX conjugates was gradient eluted with mobile phases of acetonitrile and water at a flow rate of 1.0 ml/min. The detailed gradient elution condition was: 0→10 min 60% acetonitrile, 10→30 min 100% acetonitrile, 30→40 min 60% acetonitrile. The column temperature was set at 40℃ and the detection wavelength was set at 227 nm.


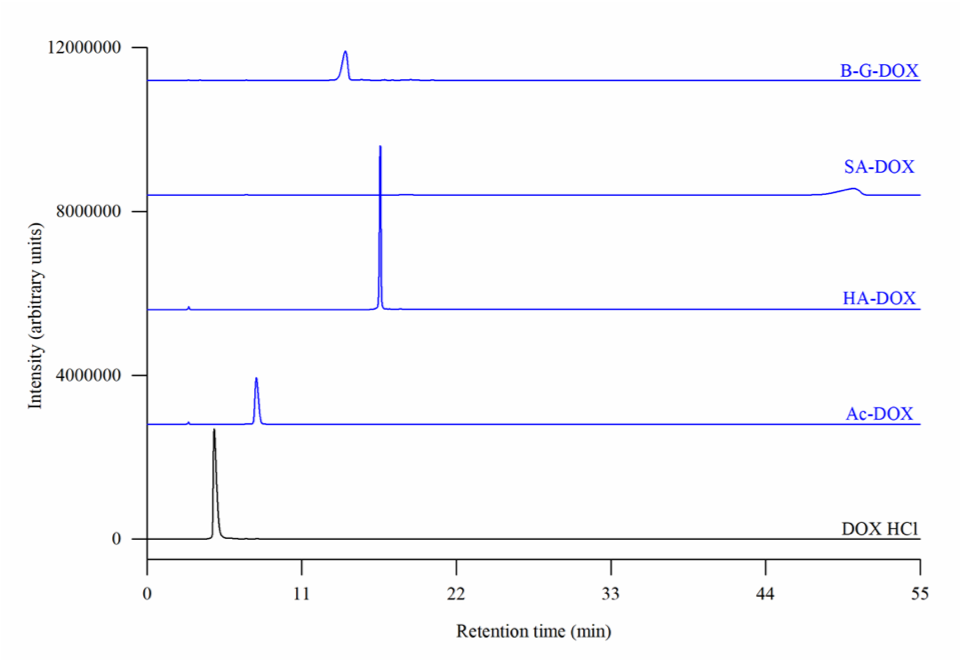


**Figure S5. HPLC chromatogram of DOX conjugates.** The DOX conjugates was gradient eluted with mobile phase A (methanol: 10mM phosphate-0.4% triethylamine buffer = 45: 55) and mobile phase B (methanol: 10mM phosphate-0.4% triethylamine buffer = 90: 10) at a flow rate of 1.0 ml/min. The detailed gradient elution condition was: 0→10 min 100% mobile phase A, 10→22 min 50% mobile phase A, 22→50 min 0% mobile phase A, 50→55 min 100% mobile phase A. The column temperature was set at 40℃ and the detection wavelength was set at 233 nm.


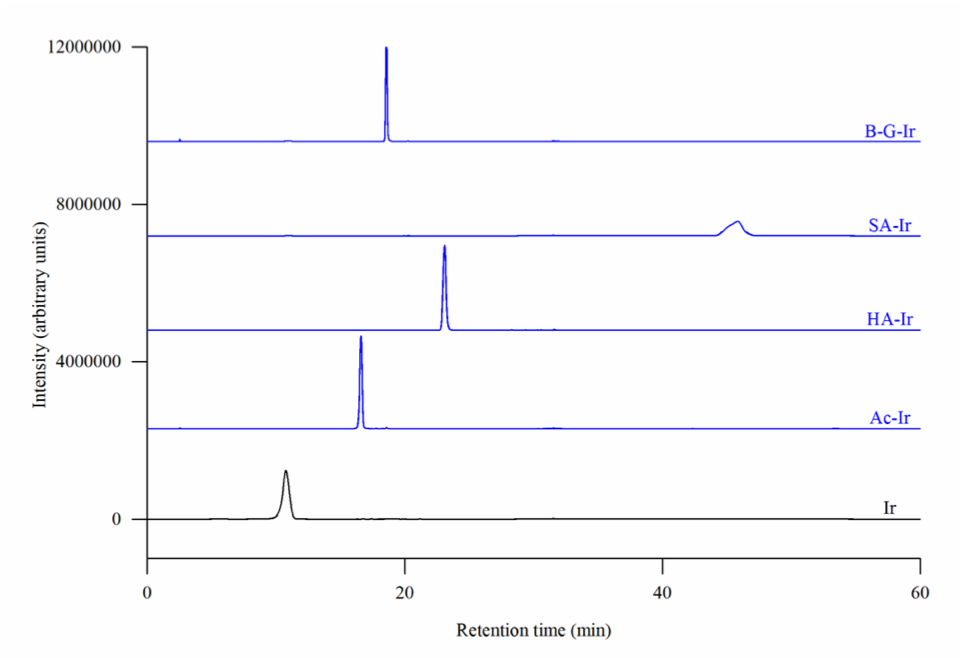


**Figure S6. HPLC chromatogram of Ir conjugates.** The Ir conjugates was gradient eluted with mobile phase A (methanol: 10mM phosphate-0.4% triethylamine buffer = 45: 55) and mobile phase B (methanol: 10mM phosphate-0.4% triethylamine buffer = 90: 10) at a flow rate of 1.0 ml/min. The detailed gradient elution condition was: 0→10 min 50% mobile phase A, 10→55 min 0% mobile phase A, 55→60 min 50% mobile phase A. The column temperature was set at 40℃ and the detection wavelength was set at 254 nm.

**X-ray diffraction (XRD)**

**
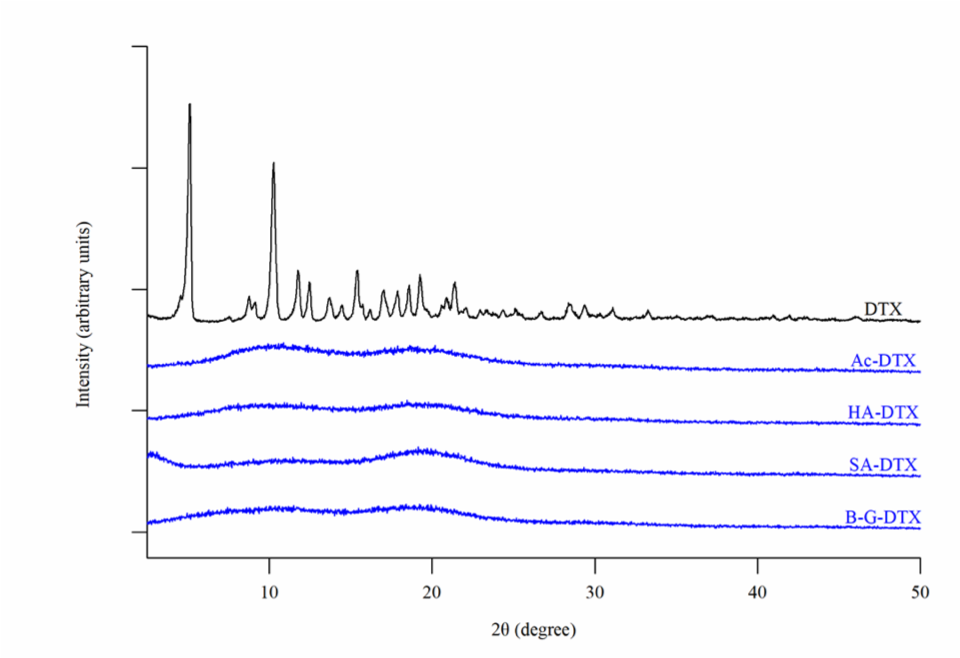
**

**Figure S7.** X-ray diffraction (XRD) spectra of DTX conjugates.

**
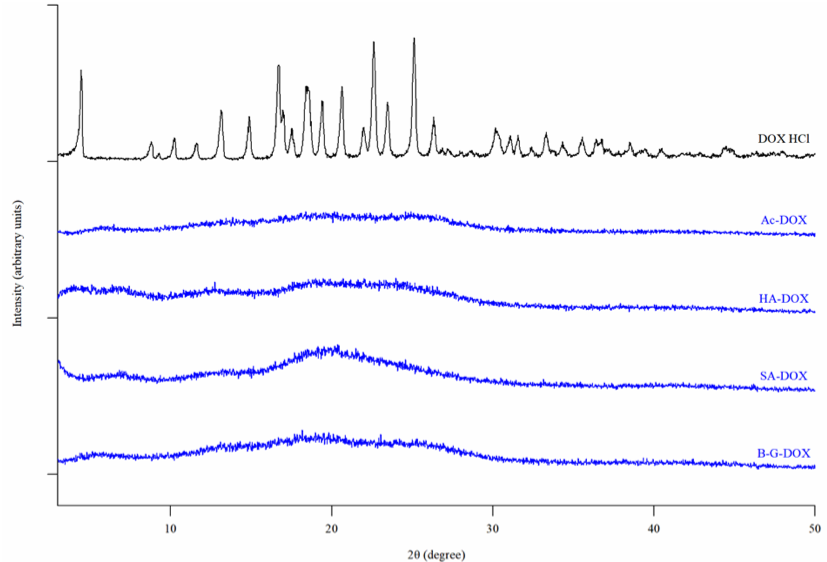
**

**Figure S8.** X-ray diffraction (XRD) spectra of DOX conjugates.

**
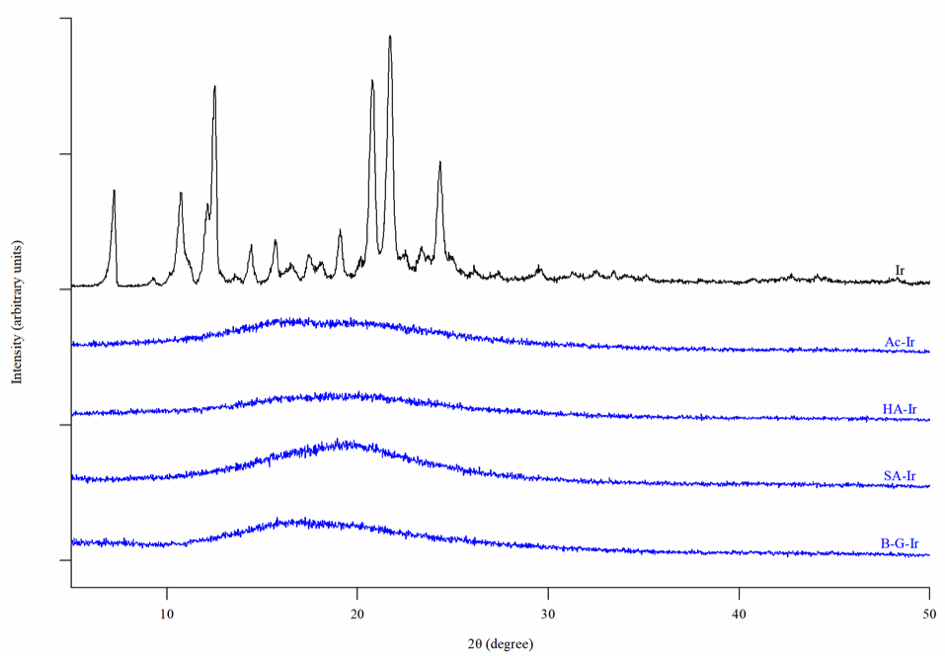
**

**Figure S9.** X-ray diffraction (XRD) spectra of Ir conjugates.
